# Properties of nanocones formed on a surface of semiconductors by laser radiation: quantum confinement effect of electrons, phonons, and excitons

**DOI:** 10.1186/1556-276X-6-582

**Published:** 2011-11-07

**Authors:** Artur Medvid, Pavels Onufrijevs, Alexander Mychko

**Affiliations:** 1 Research Laboratory of Semiconductor Physics, Institute of Technical Physics, Riga Technical University, 14/24 Azenes Str., Riga, LV-1048, Latvia

## Abstract

On the basis of the analysis of experimental results, a two-stage mechanism of nanocones formation on the irradiated surface of semiconductors by Nd:YAG laser is proposed for elementary semiconductors and solid solutions, such as Si, Ge, SiGe, and CdZnTe. Properties observed are explained in the frame of quantum confinement effect. The first stage of the mechanism is characterized by the formation of a thin strained top layer, due to redistribution of point defects in temperature-gradient field induced by laser radiation. The second stage is characterized by mechanical plastic deformation of the stained top layer leading to arising of nanocones, due to selective laser absorption of the top layer. The nanocones formed on the irradiated surface of semiconductors by Nd:YAG laser possessing the properties of 1D graded bandgap have been found for Si, Ge, and SiGe as well, however QD structure in CdTe was observed. The model is confirmed by "blue shift" of bands in photoluminescence spectrum, "red shift" of longitudinal optical line in Raman back scattering spectrum of Ge crystal, appearance of Ge phase in SiGe solid solution after irradiation by the laser at intensity 20 MW/cm^2^, and non-monotonous dependence of Si crystal micro-hardness as function of the laser intensity.

## 1. Introduction

Many experimental and theoretical investigations exist on heterostructures of self-assembled nanocones, e.g., Ge/Si [1], InAs/GaAs [2]. Usually nanocones are considered as quantum dots (QDs)--QD quantum system, with a condition ratio diameter/height of nanocones is equal 1. If solid angle α at top is > 60°, then the nanocone transforms into a quantum well (QW)--2D quantum system, due to large diameter of nanocones in comparison with the height and quantization of energy of particles (e.g., excitons) takes place only in vertical direction [2]. The decrease of nanocones' solid angle α < 60° leads to fundamental changes of its properties. QD transforms into a quantum wire (QWi)--1D quantum system with gradually decreasing diameter from the base till the tip of the cone. The last one is a unique system which has wide technical applications, for example, 1D-graded bandgap structure in elementary semiconductor [3]. It is possible to form these two types of quantum systems by laser radiation (LR).

Photo-thermo-deformation model [4] has been proposed for explaining self-assembly of nanostructures on a surface of a semiconductor by LR. According to this model, conversion of light into heat and lateral deformation of the crystalline lattice of a semiconductor takes place due to inhomogeneous absorption of light, leading to formation of periodical structure on the surface due to redistribution of point defects (interstitials and vacancies).

Nanostructures, such as, QD, QWi, and QW, are formed in semiconductors by widely used methods, e.g., molecular beam epitaxy (MBE) [5], ion implantation [6], chemical vapor deposition [7], laser ablation [8]. By these methods, nanostructures mostly grow in random manner, and parameters of such materials are not controlled, it is the so-called self-assembly manner [9].

In this article, possibilities to control parameters of nanocones, such as height and distribution, on the surface of a semiconductor by the Nd:YAG laser intensity, wavelength, and pulse duration have been proposed. Considering quantization of quasi-particles (e.g., excitons, phonons, etc.) in nanocone is a special case, since diameter of nanocone is a monotonous function of height, leading to gradual change of bandgap. Graded bandgap structure has an effect on properties of particles and quasi-particles, such as mobility and intrinsic concentration of electrons and holes, energy of excitons, phonons, and plasmons. Therefore, study of nanocones' formation mechanism and nanocones' properties is an important task for future nanoelectronics and optoelectronics industry.

## 2. Materials and methods

Ge (100) i-type single crystal samples with sizes 10.0 × 5.0 × 5.0 mm^3 ^and resistivity ρ = 45 Ωcm were used in experiments. The samples were polished mechanically and etched in CP-4A (mixture of 16% of HF; 64% of HNO_3 _and 20% of CH_3_COOH) solution to ensure minimal surface recombination velocity *S*_min _= 100 cm/s on all the surfaces. Commercial *p*- and *n*-type Si(100), (111) single crystals were investigated in the experiments. Solid solution of SiGe, containing 30% of Ge atoms (Si_0.7_Ge_0.3_), grown by MBE on top of a 150-nm thick Si buffer layer on Si was studied in the experiments. High-purity solid solution of CdZnTe, containing 10% of Zn atoms (Cd_0.9_Zn_0.1_Te), grown by high-pressure vertical zone melting method was used in the experiments as well. The grown crystals were cut into 10.0 × 10.0 × 1.0 mm^3 ^wafers. SiO_2 _protective layer on the irradiated surface of the samples was applied in the experiments with Si and Cd_0.9_Zn_0.1_Te for preventing oxidation of Si nanocones and evaporation of Cd atoms from Cd_0.9_Zn_0.1_Te surface.

Radiation by fundamental frequency of a pulsed Nd:YAG laser for Ge single crystals and Si_0.7_Ge_0.3 _solid solution with following parameters was used: wavelength λ_1 _= 1064 nm, pulse duration τ = 15 ns, pulse repetition rate 12.5 Hz, power *P *= 1.0 MW. For Si and Cd_0.9_Zn_0.1_Te single crystals, the second harmonic of the laser with λ_2 _= 532 nm and τ = 10 ns was applied. Laser beam to the irradiated surface of the samples was directed normally. The spot of laser beam of 3 mm diameter was scanned over the sample surface using a two-coordinate manipulator with 20 μm step. All experiments of nanocones' formation were performed in ambient atmosphere at pressure of 1 atm, *T *= 20°C, and 60% humidity.

The surface morphology by atomic force microscope (AFM) was studied. Optical properties of non-irradiated and irradiated samples by photoluminescence (PL) and back scattering Raman methods were investigated. For PL, the 488-nm line of a He-Cd laser and for Raman back scattering an Ar^+ ^laser with λ = 514.5 nm were used. Measurement of the PL spectra for Si, Ge, and SiGe solid solution at room temperature was performed, but for solid solution of CdZnTe--at 5°K. Detailed description of these experiments is published in the following articles: for elementary semiconductors Ge [10] and Si [3] and solid solutions Si_0.7_Ge_0.3 _[11] and Cd_0.9_Zn_0.1_Te [12]. Microhardness test was performed using a microhardness tester PMT-3 (manufactured by LOMO in USSR) using indentation method with original self-adjusting loading device, allowing to carry out precision microhardness measurements at very small test loading. The indenter was used a Vicker's diamond pyramid and relaxation time was 15 s. Each point in the figures corresponds to 20 measurements of processed statistically.

## 3. Results and discussion

The mechanism of nanocones' formation on the irradiated surface of Si_0.7_Ge_0.3 _solid solutions is characterized by two stages--laser redistribution of atoms (LRA) and selective laser annealing (SLA) [13].

The first stage, LRA, is characterized by formation of heterostructures such as Ge/Si due to drift of Ge atoms toward the irradiated surface of the sample in the gradient of temperature, the so-called thermogradient effect (TGE) [14]. This process is characterized by positive feedback: after every laser pulse, the gradient of temperature increases due to the increase of Ge atoms' concentration at the irradiated surface. New Ge phase is formed at the end of the process. Ge atoms are localized at the surface of Si like a thin film. As a result, LRA stage gradually transits to SLA stage.

The second stage, SLA, is characterized by formation of nanocones on the irradiated surface of a semiconductor by selective laser heating of the top layer with following mechanical plastic deformation of the layer as a result of relaxation of the mechanical compressive stress arising between these layers due to mismatch of their crystal lattices and selective laser heating. SLA occurs due to higher absorption of the LR by the top layer than the buried layer.

**Figure 1:**
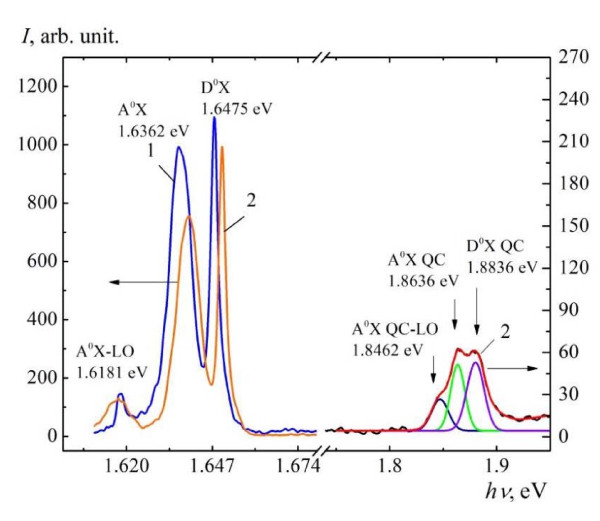
**PL spectra of the Cd_0.9_Zn_0.1_Te measured at temperature 5 K: curve 1, non-irradiated; curve 2, irradiated by the laser at *I *= 12.0 MW/cm^2^**.

A similar two-stage's mechanism can be used for nanocones' formation by laser beam on ternary solid solution Cd_0.9_Zn_0.1_Te. Irradiation of the Cd_0.9_Zn_0.1_Te solid solution by the laser leads to the drift of Cd atoms toward irradiated surface and of Zn atoms--in the bulk of the semiconductor due to TGE [14]. As a result, formation of CdTe/Cd_1-*x*1_Zn*_x1_*Te heterostructure, where *x*_1 _> 0.1, takes place. Decrease of Zn atoms' concentration in the top layer with intensity of LR, according to the proposed model, leads to the "red shift" of the exciton bands in PL spectra, as was shown in [12], but increase of the Zn atoms' concentration in buried CdZnTe layer manifests in "blue shift" of the PL spectrum, as shown in Figure 1 on the left side. These effects do not compensate each other since they take place in different layers. Of course, it is possible to observe both PL spectra simultaneously at intermediate situation. Exactly such situation is observed in PL spectrum, in Figure 1, after destruction of the CdTe top layer and formation of nanocones on the irradiated surface of the sample. Relaxation of the mechanical compressive stress in CdTe layer comes to an expression as self-assembly of nanocones on the irradiated surface of the structure like Ge nanocones in SiGe solid solution. Calculation of the mechanical compressive stress in CdTe top layer using the maximum of the "blue shift" of excitons bound to shallow neutral acceptors (A^0^X) exciton band from Figure 1 and dE_g_/*dP *= 10 eV/Pa [10], where *E*_g _and *P *are bandgap of CdTe crystal and mechanical stress, respectively, gives *P *= 4.62 × 10^5 ^Pa. This value corresponds to the ultimate strength limit of CdTe [11]. Calculation of QD diameter using the formula from [12] and the "blue shift" of A^0^XQC in PL spectrum on 0.27 eV give diameter of the QDs up to 10.0 nm. These data correspond to the size of nanocones (height and diameter of the bottom of the cones are about 10 nm with an error of ± 1 nm) measured using 3D image of AFM. An evidence of presence of the exciton quantum confinement in nanocones is the decrease of longitudinal optical (LO) phonon energy by 0.7 meV in PL spectrum (as can be seen from Figure 1, positions of A^0^X-LO and A^0^XQC-LO zero phonon bands), that is the so-called phonon quantum confinement effect [13]. Our calculation on Zn atom's distribution depending on intensity of LR using the thermo-diffusion equation has shown that the process of CdTe/Cd_1-*x*1_Zn*_x1_*Te heterostructure formation is characterized by gradual increase of Zn atom's concentration in the buried layer with intensity of LR up to 8%. It means concentration of Zn atoms is 0.18. The thickness of the CdTe layer after irradiation by the laser with intensity of *I *= 12.0 MW/cm^2 ^becomes 10 nm.

Moreover, the stress is caused by both due to large lattice mismatch between CdTe and Cd_1-*x*1_Zn*_x1_*Te layers [15] and SLA stage. Relaxation of the mechanical compressive stress in CdTe layer as a result of nanocones formation on the irradiated surface of Cd_0.9_Zn_0.1_Te sample similar to Stransky-Krastanov' mode [16] takes place. Appearance of a new exciton band at 1.872 eV in PL spectrum of Cd_0.9_Zn_0.1_Te sample at higher intensity of LR was observed. Reconstruction of this band (see Figure 1 on the right side) shows that it consists of three lines which look like A^0^X, D^0^X (excitons bound to shallow neutral donors) and A^0^X-LO (phonon replica of excitons bound to shallow neutral acceptors) lines in the non-irradiated PL spectrum of the structure. Therefore, we connect both the new band appearance in PL spectrum and the nanocones' formation on the irradiated surface of the semiconductor with exciton quantum confinement in nanocones and denote them as A^0^XQC and D^0^XQC lines.

In the case of the elementary semiconductors, at the first stage of the process, a thin top layer with mechanical compressive stress due to separation and redistribution of interstitials and vacancies in gradient temperature field [14] on the irradiated surface of the semiconductors is formed. As a result, interstitials are concentrated at the irradiated surface of semiconductor, forming the top layer. Vacancies are concentrated under the top layer forming a buried layer with mechanical tension due to the absence of atoms. Sometimes vacancies form nanocavities [17]. At the second stage of the process, nanocones are formed on the irradiated surface of the semiconductors due to plastic deformation of the top layer in the same way as in the previous case with semiconductor solid solutions.

**Figure 2:**
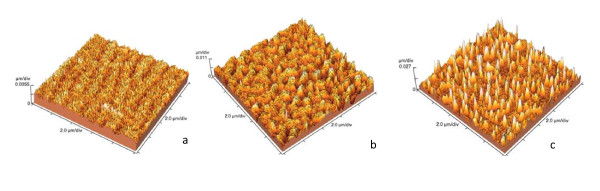
**AFM images of irradiated Si_0.7_Ge_0.3 _solid solution**. AFM images of Si_0.7_Ge_0.3 _surfaces irradiated by the Nd:YAG laser at intensity **(a) **2.0 MW/cm^2^; **(b) **7.0 MW/cm^2 ^and **(c) **20.0 MW/cm^2^.

**Figure 3:**
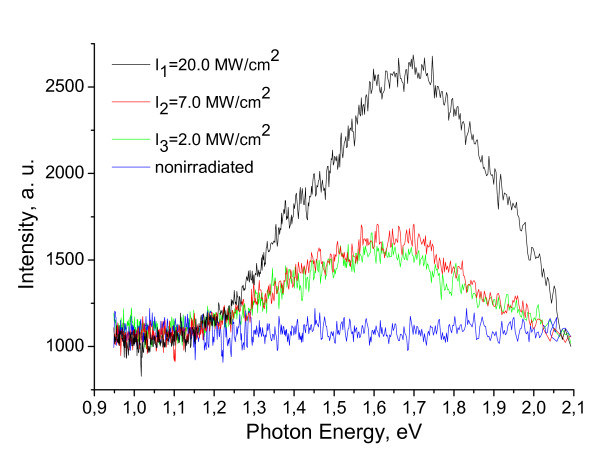
**PL spectra of Si_0.7_Ge_0.3 _solid solution: nonirradiated and irradiated by Nd:YAG laser**.

**Figure 4:**
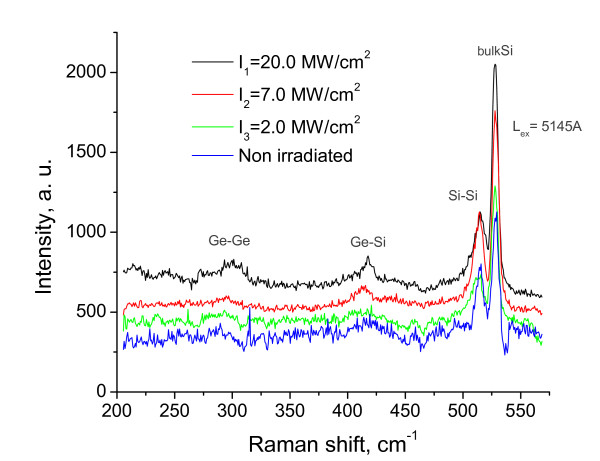
**Back scattering Raman spectra of Si_0.7_Ge_0.3 _solid solution: non-irradiated and after irradiation by the laser**.

**Figure 5:**
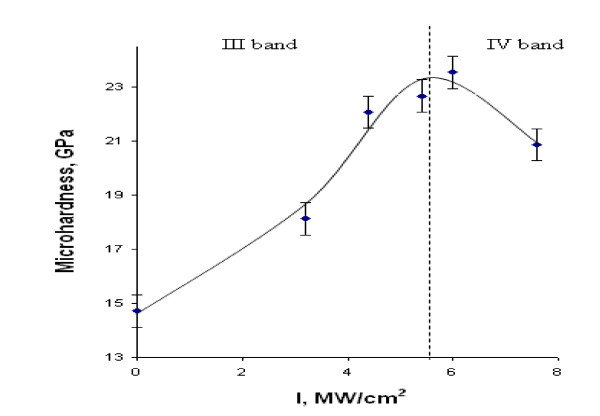
**Microhardness of *n*-Si (111) wafer depending on laser intensity at load on indenter 20 g**.

To approve two-stage mechanism of nanocones formed on the semiconductor surface, we have proposed several evidences:

1. Appearance of nanocones on the irradiated surface of semiconductors and their height dependence to the laser intensity has been found by measurements of the irradiated surface morphology by AFM, as shown in Figure 2.

2. The "blue shift" of the PL spectra and increase of PL bands' intensity of Si, Ge, and Si_0.7_Ge_0.3 _crystals with increase of the LR intensity due to quantum confinement effect, as shown in Figure 3 for Si_0.7_Ge_0.3 _crystal, is the next evidence of the SLA stage.

3. The presence of the first stage is appearance and increase of intensity of LO phonon line with frequency 300 cm^-1 ^in Raman back scattering spectrum of Si_0.7_Ge_0.3 _solid solution after irradiation by the laser. A new Ge phase is observed on the irradiated surface of Si_0.7_Ge_0.3 _[18], as shown in Figure 4.

4. Non-monotonous dependence of Si crystal microhardness as a function of the laser intensity. The increase of microhardness with increasing LR intensity is explained by formation of mechanically compressed layer at the irradiated surface due to increase of concentration of the interstitial atoms of Si at the surface in temperature gradient field, which is characteristic to the LRA stage. The decrease of the microhardness is explained by formation of nanocones as a result of plastic deformation of the mechanically stressed layer, which is characteristic to the SLA stage, as shown in Figure 5.

5. The shift of PL spectrum of Cd_0.9_Zn_0.1_Te solid solution at low intensity of the LR toward lower energy of quantum--the "red shift" [12]--is the next evidence of the first stage of thin CdTe layer formation. The shift of bands in PL spectrum of Cd_0.9_Zn_0.1_Te solid solution at high intensity of the LR toward higher energy of quantum--the "blue shift" [12] and appearance of a new PL band at higher energy of quantum--exciton quantum confinement effect, as shown in Figure 1, are evidences of the second stage of the mechanism.

## 5. Conclusions

1. For the first time we have shown a possibility to form 1D-graded bandgap structure in elementary semiconductor. The graded bandgap is formed in nanocones due to quantum confinement effect.

2. We have shown the possibility to control nanocones' features by changing LR parameters, such as intensity, wavelength, and pulse radiation duration.

3. The new PL band at 1.8718 eV is observed after irradiation of Cd_0.9_Zn_0.1_Te solid solution by Nd:YAG laser at intensity 12.0 MW/cm^2^. The origin of this PL band we connect with exciton quantum confinement effect in nanocones was formed on the irradiated surface of the semiconductor.

## Abbreviations

A^0^X: excitons bound to shallow neutral acceptors; A^0^X-LO: longitudinal optical (LO)-phonon replica of excitons bound to shallow neutral acceptors; AFM: atomic force microscopy; D^0^X: excitons bound to shallow neutral donors; LR: laser radiation; LRA: laser redistribution of atoms; MBE: molecular beam epitaxy; PL: photoluminescence; QD: quantum dots; QWi: quantum wires; QW: quantum well; SLA: selective laser annealing; TGE: thermogradient effect.

## Competing interests

The authors declare that they have no competing interests.

## Authors' contributions

AM conceived the studies and coordinated the experiment. All of the authors participated to the analysis of the data and wrote the article. AMy carried out the sample preparation, the measurements for solid solutions of CdZnTe. PO carried out the sample preparation, the measurements for elementary semiconductors: Si, Ge and solid solution of SiGe. All the authors read and approved the manuscript.
